# Perovskite Nanoparticles Toxicity Study on Airway Epithelial Cells

**DOI:** 10.1186/s11671-018-2845-2

**Published:** 2019-01-08

**Authors:** Shih-Ming Tsai, Maria Mesina, Tyler Goshia, Meng-Hsuen Chiu, Julia Young, Angelo Sibal, Wei-Chun Chin

**Affiliations:** Bioengineering Program, School of Engineering, University of California at Merced, 5200 North Lake RD, Merced, CA 95343 USA

**Keywords:** Nanoparticles, Perovskite, Ca^2+^ signal, ROS, Apoptosis

## Abstract

Research on the toxicity of nanoparticles has developed over recent years due to their increasing prevalence in common everyday materials. Various nanoparticles have been reported to promote and induce mucus secretion, which could potentially lead to airway damages and respiratory complications. Lanthanum strontium manganite (LSM) is a nanoparticle widely used in solar oxidized fuel cells (SOFCs) due to its high electrical conductivity, high electrochemical activity for O_2_ reduction reaction, high thermal stability and compatibility of SOFC electrolytes, and most importantly, its microstructural stability and long-term performance. Very few studies have been conducted on LMS’s toxicity, thus its effect on airway cells was investigated in this study. After treating trachea cells with increasing concentrations of LSM ranging up to 500 μg/ml, we found that it has a moderate effect on cell viability, ROS production, cytochrome C, and caspase 3 expression. Despite its minimal impact on stated apoptosis-inducing characteristics, LSM illustrated an inhibiting effect on mucus secretion. We obtained a decreasing trend in mucus secretion with an increased concentration of the LSM treatment. Overall, LSM’s advancement in SOFCs necessitated a toxicity study, and although it does not show a significant toxicity to trachea cells, LSM reduces mucus secretion, and can potentially interfere with airway clearance.

## Introduction

Lanthanum strontium manganite (LSM) is a nanoparticle equipped with a perovskite-based crystal structure. It takes the general form of “ABO3,” where lanthanum and strontium are in the A site and manganese in the B. This leads to its general formula of La_1 − x_Sr_x_MnO_3_, in which x stands for the strontium doping levels that is dependent upon the application of the nanoparticle. LSM can be used in a powdered form as tape casting, air/thermal/plasma spray, and for fuel cell applications [1–4].

In the recent effort to reduce pollution and create a hydrogen-based economy, much research has been focused on solid oxide fuel cells (SOFCs) [5, 6]. In turn, LSM perovskites have attracted research attention due to its significant role in SOFCs as one of its most important electrode materials [4, 7]. LSM nanoparticles are spherical high surface metal particles that appear as a brown or black crystalline powder. Although numerous studies on LSM’s mechanical and electrical properties have been conducted [1, 3, 8, 9], very little research has focused on its biological effects [10–12]. Numerous nanoparticles have illustrated tendencies to promote mucus secretion and accumulation, thus are linked to respiratory illnesses [13, 14]. Research upon LSM nanoparticles shows promising ideas for biomedical applications [15–17]. Recently, research on LSM has shown its potential for anti-cancer therapy [12, 18, 19]. With the correct synthesis procedures and surface modifications, LSM has the ability to be a MRI contrast and hyperthermia agent as well as a drug carrier [20–22]. However, the possibility of toxicity should be assessed prior to further medical applications. There are only limited studies on the toxicity effect of perovskite [10, 23], and there is no significant toxicity effect that had been reported so far.

In this study, we investigated the toxicity of LSM nanoparticles, while being exposed to primary tracheal epithelial cells to determine the toxicity concentration range. We then analyzed the reactive oxygen species (ROS) production and mitochondria damage along with the mucus secretion response with fluorescent microscopy [24]. The level of apoptosis progression with or without LSM nanoparticles was examined with cytochrome C and caspase 3 assays [25].

## Results and Discussions

### NPs Characterization and Cell Viability Assay

**Fig. 1:**
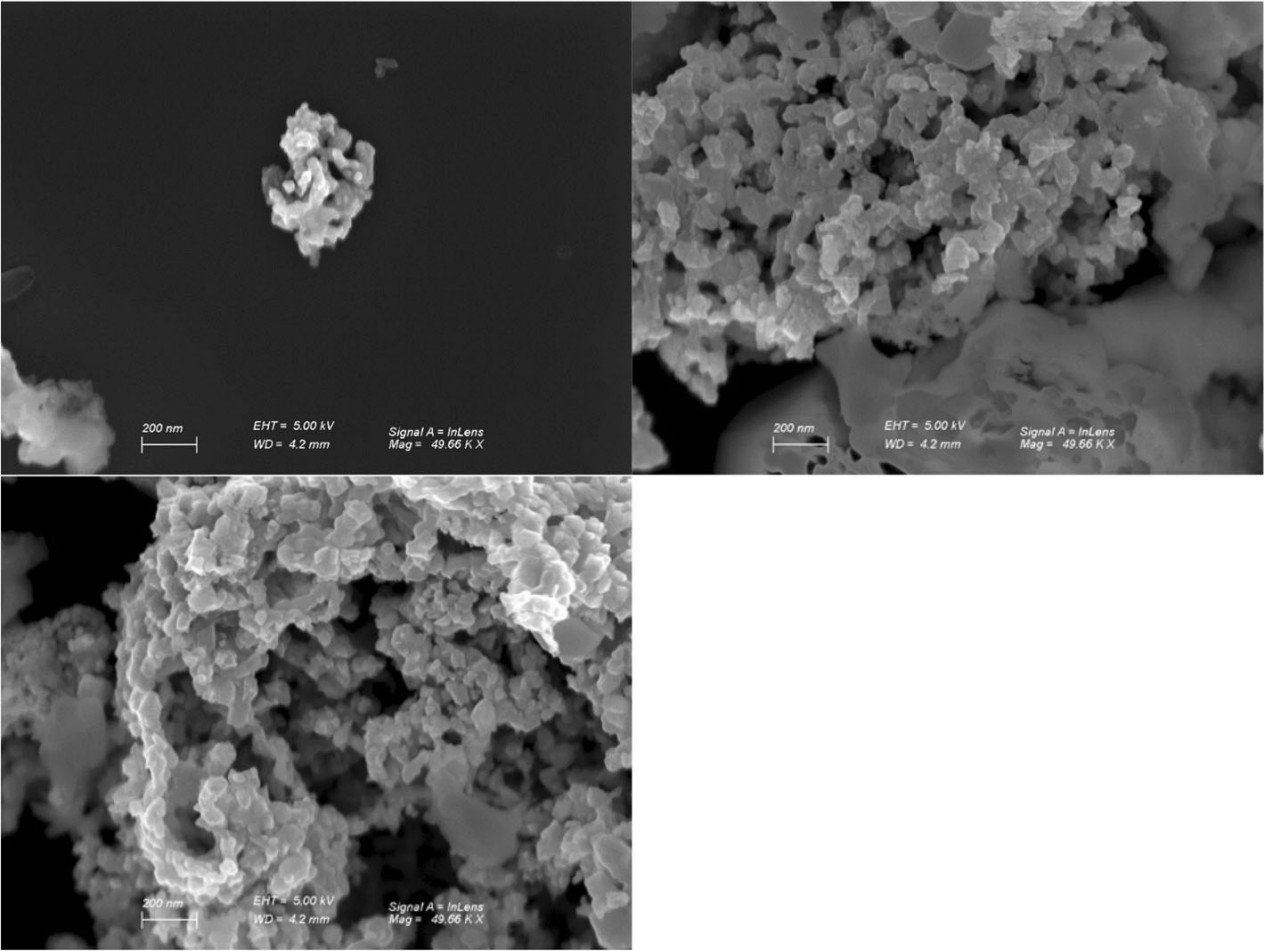
SEM image of LSM physical characteristics. The single particle size was around 35 nm in diameter and the aggregation of LSM size varied from 200 nm to a few μm

The toxicity of nanoparticles is dependent on its physical properties, such as geometry, size distribution, and surface area. Prior to the toxicity experiments, these characteristics were analyzed using SEM. The LSM nanoparticles illustrated significant surface coarseness and were distributed in aggregates of various sizes, approximately 35 nm to 200 nm in diameter (Fig. 1).

**Fig. 2:**
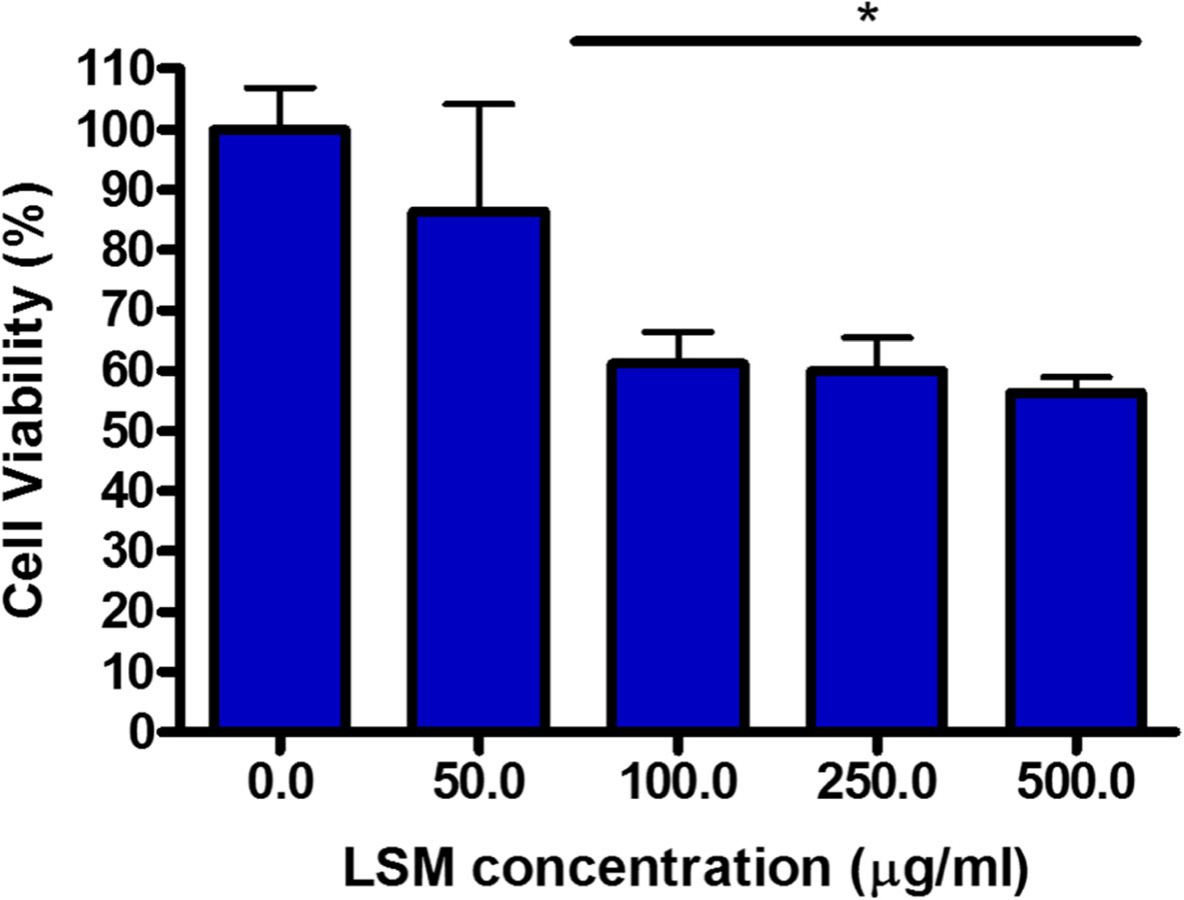
Trachea cell viability after increasing LSM concentration, shown here as Log[ng/ml]. The colometric CCK8 cell viability assay was used to measure, and its average per concentraion increment was calculated. (*n* > 6)

We have conducted numerous toxicity experiments on trachea airway cells with a variety of NPs in our previous study [26, 27], therefore this cell line was chosen for this biological study. In order to assess the overall toxicity of LSM on trachea cells, the colorimetric CCK8 cell viability assay was performed [25, 27]. As seen in Fig. 2; cell viability assay, there was a dramatic change in population occurring from 50 to 100 μg/ml concentrations of LSM. However, at LSM concentrations greater than 100 μg/ml, the population maintained at a relatively steady range, showing no significant changes.

### Production of ROS and Mucin Release

**Fig. 3:**
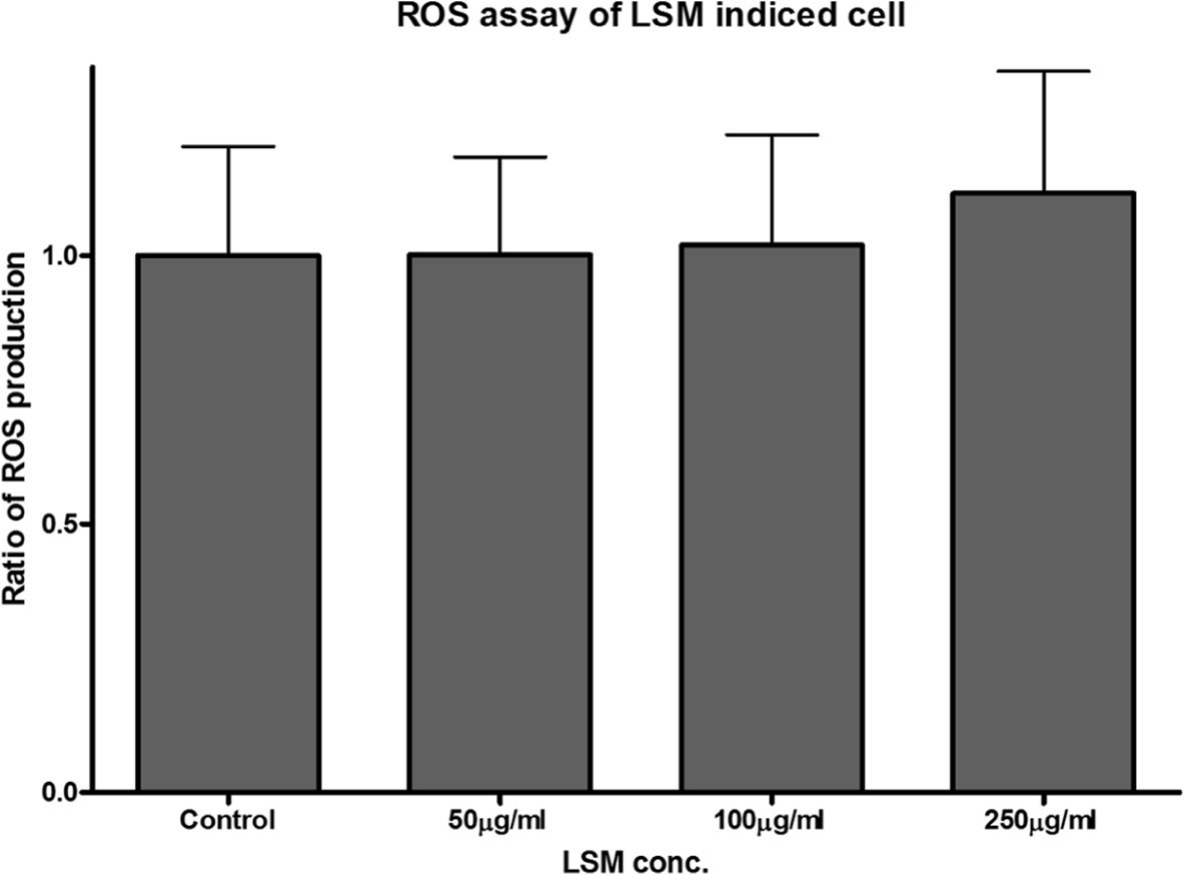
ROS production of trachea cells after increasing LSM concentrations. Ratios of ROS production per each LSM concentration treatment were calculated by compared to the control group. (*n* > 100)

ROS production instigates apoptosis, thus contributes to nanoparticle cytotoxicity. According to Fig. 3; ROS production, increasing the concentration of LSM does not greatly affect ROS production. The 250 μg/ml LSM concentration differed the most from the control, but its deviation was not significant, indicating that LSM has no remarkable effect on ROS production.

**Fig. 4:**
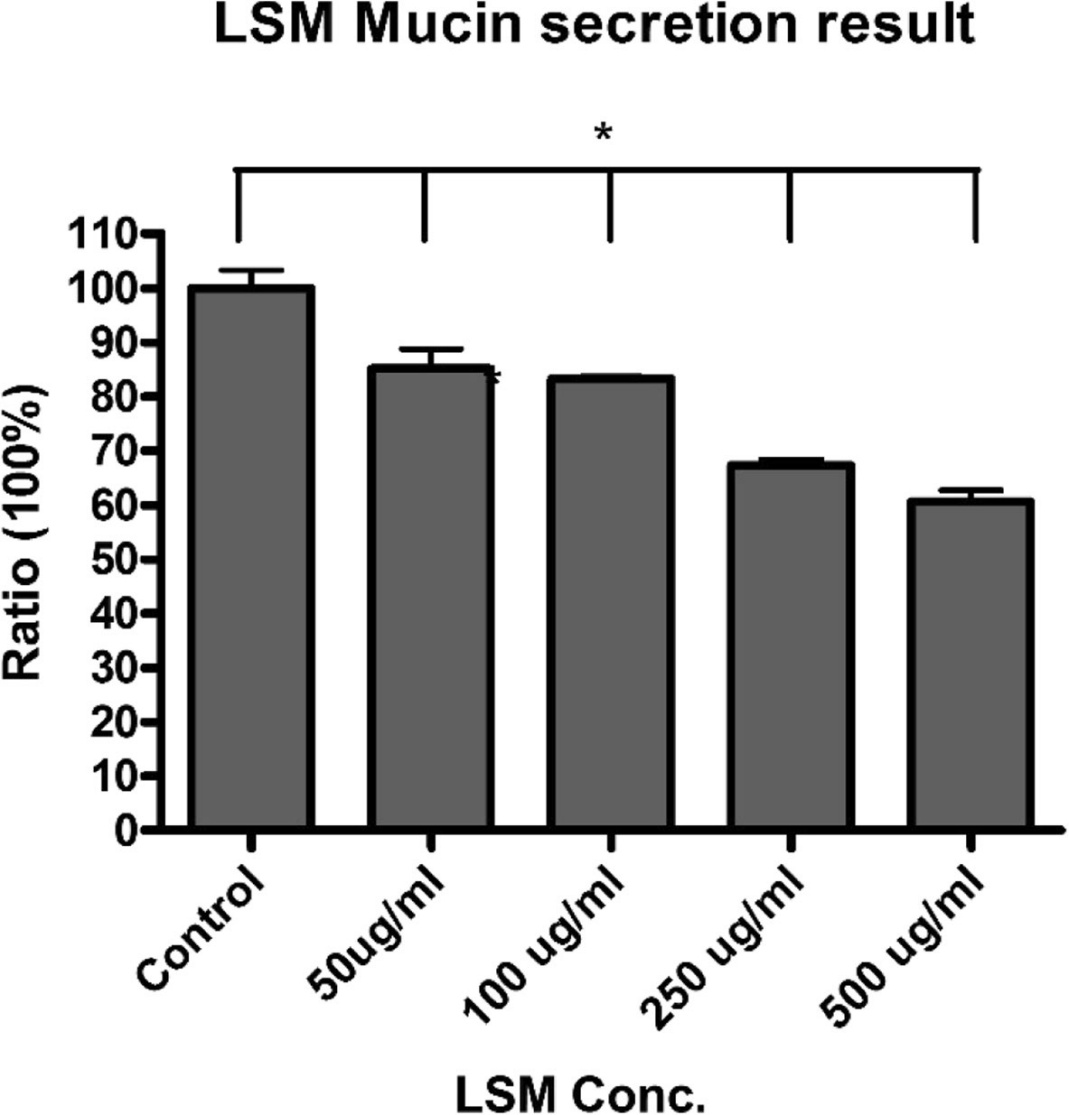
Mucin secretion results after 15-min treatments of increasing LSM concentrations. The ratios were calculated for each LSM concentration by comparing it to the control group. Assessment of cell surface mucin secretions was done by ELLA (enzyme-linked lectin assay). (*: *n* > 6, *p* < 0.05)

From Fig. 4; mucus bata, after a 15-min treatment, there was no significant effect on cell viability, and as LSM concentrations increased, mucin secretion decreased. The release of mucin was reduced as low as 40% when cells were treated with 500 μg/ml of LSM, and could potentially be reduced further with even higher concentrations of LSM. This reduction suggests that LSM has an inhibitory effect on mucus release.

### Mitochondrial Damage and Apoptosis Processes

**Fig. 5:**
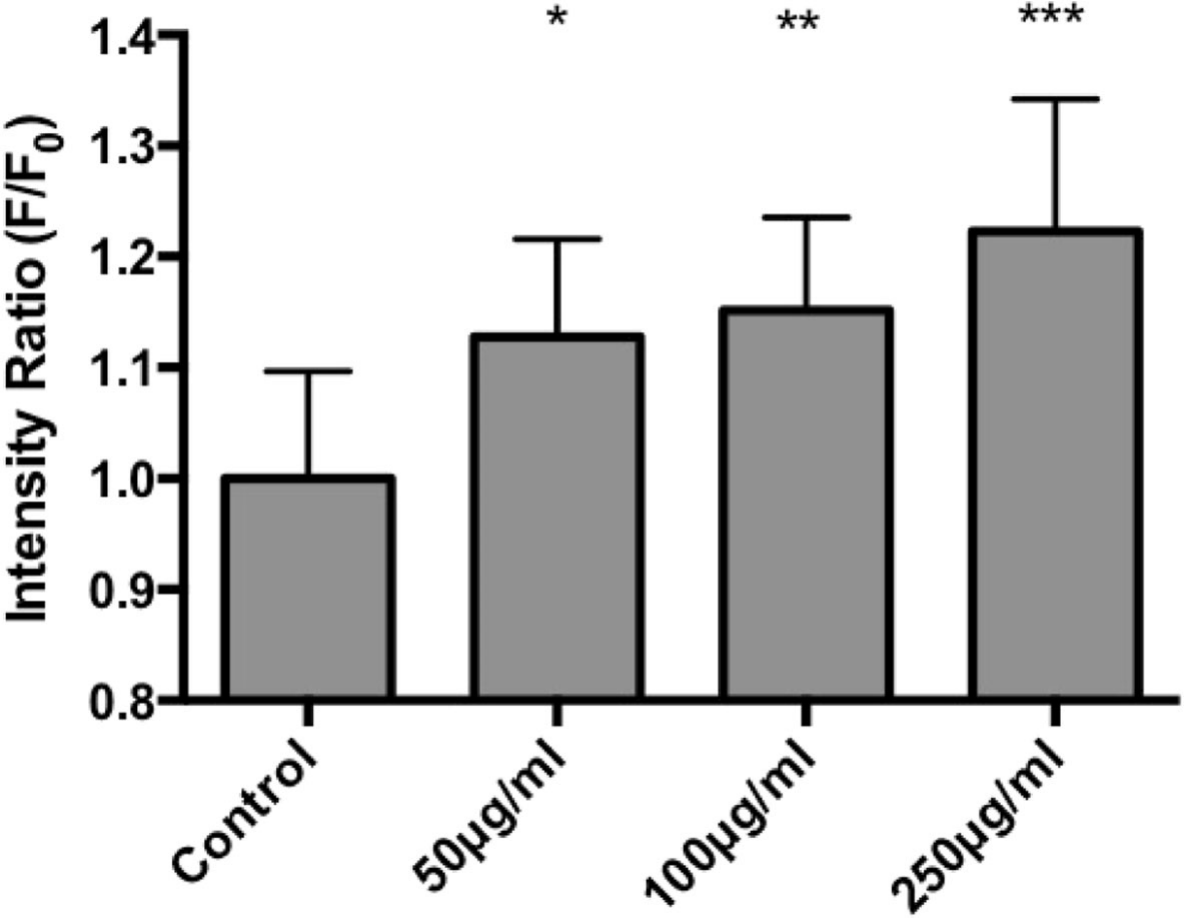
Fluorescent intensity results of JC-1 indicator dye in four different conditions of treatment on trachea cell to indicate mitochondrial integrity. (*, **, ***: *n* > 100, *p* < 0.05)

The early stage of apoptosis is typically represented by mitochondrial damage. To analyze this phenomenon, the JC-1 dye was used as a mitochondrial membrane potential indicator. The intensity ratio induced by the JC-1 dye correlates with the loss of mitochondrial integrity, and according to Fig. 5, as LSM concentrations were increased, mitochondrial damage increased significantly after 100 μg/ml of LSM treatment. Although the results show that the mitochondria damage is significant after 100 μg/ml of LSM treatment, the caspase3 and cytochrome C shows slight decreases, as detailed below. This result suggests that LSM could cause mitochondria damage but the cells still have the capability to remain at a low-level in apoptosis progression and reduce the apoptosis marker expression.

**Fig. 6:**
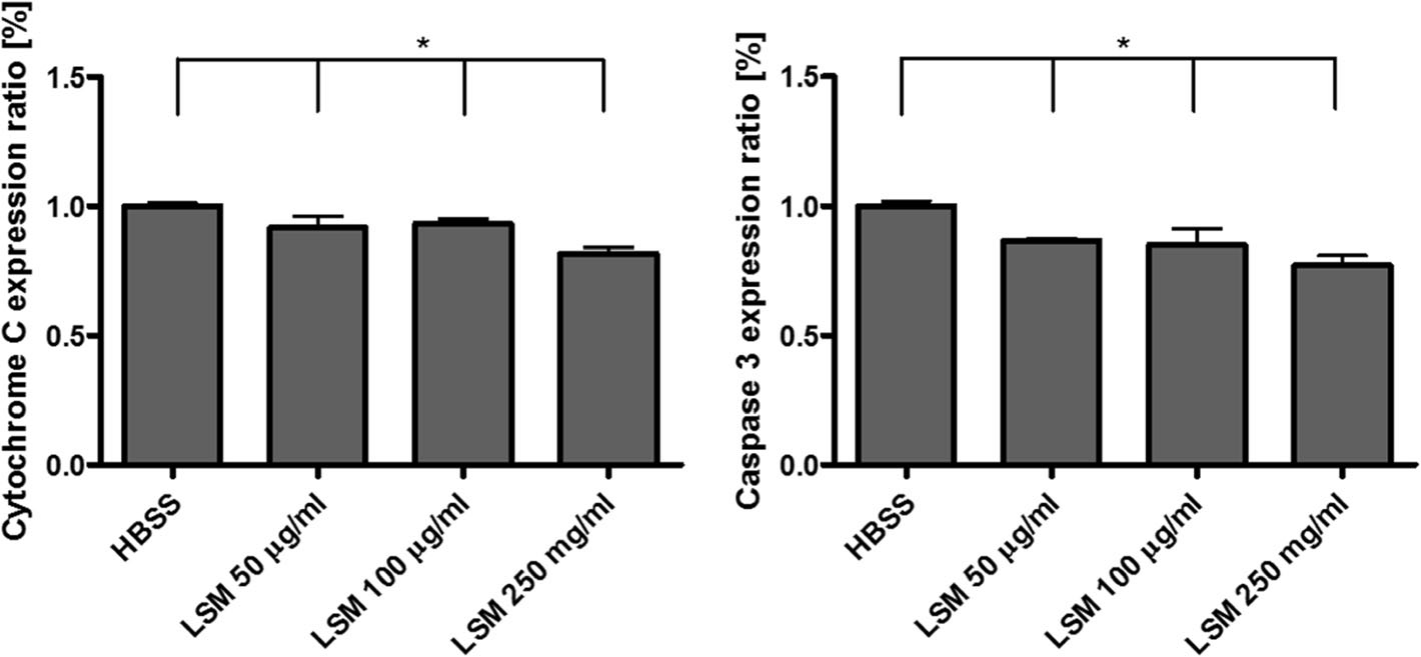
Measured apoptosis results through **a** cytochrome C expression, **b** caspase 3 expression. The ratios were obtained per LSM concentration treatment by compared to the control (HBSS) group. (*: *n* > 100, *P* < 0.05)

Apoptosis can also be measured through the expression of cytochrome C and caspase 3. As shown in Fig. 6a; apoptosis result on cytochrome C, there was a moderate decrease in apoptosis rates among the different concentrations of LSM and the control group. This same trend can be seen in Fig. 6b; apoptosis result on caspase 3, indicating very minimal toxicity due to LSM. Overall, the mitochondrial damage and apoptosis results on airway trachea cells due to LSM were very low, suggesting that LSM has no significant toxic effect on trachea epithelial cells.

## Conclusions

Recent nanotoxicity studies have attracted a lot of attention on the toxic effects of NPs due to their broad use in industrial and commercial products. The major concern of nanotoxicity is due to the generation of ROS. For example, TiO_2_ NPs are being considered as a kind of carcinogenic material due to the large amount of generated ROS that could lead to cell death and mutations [28]. There are different types of materials and compounds classified as perovskite that have been reported in recent years in a variety of different applications. This is the first study to determine the LSM toxicity on airway epithelial cells which are one of the major paths for cells to uptake NPs. This study aimed to investigate LSM’s effect on trachea cells in an effort to assess its toxicity. The results showed that LSM does not have any significant effect on apoptosis progression measured by cytochrome C and caspase 3 expressions, mitochondria integrity measured by JC-1 fluorescence, cell survival, and ROS production. However, the treatment showed a suppressing effect on mucus secretion, decreasing mucus production as LSM concentrations were increased. Ultimately, through the results obtained from this study, LSM was found to be non-toxic toward airway epithelial cells, causing no significant changes in apoptosis stages, additionally lowering mucus secretion that can jeopardize cell viability. This suggests LSM’s potential to interfere with airway mucus clearance. In our study, we demonstrated the toxicity potential for the LSM NPs and the results show that the potential risk of the toxic effect is relatively lower than other NPs that had been used for industrial and commercial application. However, further research must be done to determine whether LSM can be safely incorporated as an active ingredient in commercial solar and energy storage products.

## Materials and Methods

### Culture of Trachea Primary Cells

The trachea primary epithelial cells were isolated from normal bovine bronchial epithelium following by previously published protocols [26]. Cells were grown and maintained in serum-free medium (SFM) supplemented supplied with prequalified human recombinant epidermal growth factor 1–53 (EGF 1–53) and bovine pituitary extract (BPE) (Thermo Fisher). The primary trachea cells were cultured in collagen () pre-coated 15-cm Falcon plates and incubated in a humidified incubator at 37 °C, 5% CO_2_. Cell counts were performed using trypan blue (Sigma) exclusion and a Bright-Line Hemocytometer. Cells were passed when confluence reached 80%.

### Cell Preparation

Cells were seeded at 5 × 10^4^ cells per well in a collagen coated 96-well plate (75% confluence) for cell viability assays, 5 × 10^5^ cells per well in a collagen-coated 4-well plate (75% confluence) for Ca^2+^ signaling, ROS and mitochondria analysis. After seeding, cells were incubated for 24 h in SFM supplemented supplied with prequalified human recombinant EGF 1–53 and BPE (Thermo Fisher). Following 24 h of incubation, the medium was removed from the cells and the culture was rinsed with phosphate-buffered saline twice. PBS wash was replaced with nanoparticles sonicated in Ca^2+^ containing Hanks or Ca^2 +^ -free Hanks.

### Cell Viability Assay

Photocolorimetric determination of cytotoxicity was assessed using CCK-8 dye (Dojindo Laboratories, Tokyo, Japan) [27, 25]. CCK-8, being non-radioactive, offers a colorimetric determination of the percentage of viable cells subjected to varying NP concentrations. This assay kit measures the metabolic activity of dehydrogenases within the viable cells to convert WST-8 Tetrazolium salt into water-soluble formazan. It was prepared by adding CCK-8 in HBSS in a 1:10 dilution. Cells were rinsed with HBSS and 100 μL of the dye was loaded into each well. Then, the cells were incubated in a 37 °C, 5% CO_2_ incubator for 6 h. The absorbance was measured by using a Thermo Multiscan EX plate reader (Thermo Multiskan EX plate reader, VWR, CA, USA) at the optical density of 450 nm (650 nm reference). An average was calculated from three separate data sets for each concentration, including the untreated control, from three independent experiments and was tabulated as a percentage of the untreated control.

The cell viability was calculated by $\frac{OD_{450\mathrm{treatment}}-{OD}_{650\mathrm{treatment}}}{O{D}_{450\mathrm{control}}-O{D}_{650\mathrm{control}}}\ast 100\%$.

### Lanthanum Strontium Manganite Nanoparticle

Lanthanum strontium manganite (La_0.15_Sr_0.85_MnO_3_) (LSM) nanoparticles (35 nm, 99.5%) (Nanostructured&Amorphous Materials Inc.) was used in this study. All NP samples were sonicated before use. The concentrations used were 500 μg/ml, 250 μg/ml, 100 μg/ml, and 50 μg/ml. The range of concentrations used was decide followed the concentrations of TiO_2_ NPs found in previous reports (Dowding et al. 2014; Dowding et al. 2012; Gurr et al. 2005; Hirst et al. 2009; Niu et al. 2011). The LSM NPs were reconstituted with Hanks’ solution (Invitrogen, CA, USA) before being tested individually and sonicated for approximately 5 min immediately before use.

### Scanning Electron Microscope

LSM NPs were prepared into 5 μg/ml and drop cast on clean silicon wafer and air dried to remove residual water. The size of NPs was independently confirmed using a scanning electron microscope (Gemini SEM, Zeiss).

### Intracellular Reactive Oxygen Species Production

Reactive oxygen species (ROS) production was evaluated by fluorescence microscopy using oxidation of CM-H2DCFDA dye (Invitrogen, CA, USA) [24]. The cells (1 × 105 cells/well) were cultured for 24 h before being rinsed with PBS solution. Samples were stained by applying a loading buffer containing 2 μM reconstituted CM-H2DCFDA dye in medium for 30 min. The stained samples were washed with PBS three times and set aside for 5 min of recovery time for cellular esterases to hydrolyze the AM or acetate groups and render the dye responsive to oxidation. Hanks’ buffer, containing LSM NPs at concentrations ranging from 0 to 500 μg/ml in 50 μg/ml, was then incubated with the cells for 15 min in 37 °C followed by PBS washing. Fluorescent images of ROS generated in cells were captured and analyzed by calculating the ratio of increase in fluorescent intensity between different treatments and control groups.

### Mitochondria Damage Measurement

The mitochondrial inner trans-membrane potential was assessed using polychromatic 5,5′,6,6′-tetrachloro-1,1′,3,3′-tetraethylbenzimidoazolyl-carbocyanio iodide (JC-1 Sigma) [25]. JC-1 is a lipophilic fluorescent cation that can be incorporated into the mitochondrial membrane, where it is dependent on membrane potential state aggregates. Aggregation changes the fluorescence properties of JC-1, shifting from green to red fluorescence. Intact mitochondrial membranes stained with JC-1 exhibit a pronounced red mitochondrial fluorescence that is detectable by fluorescence microscopy. A breakdown of the mitochondrial membrane potential results in a subsequent decrease in green fluorescence and increase in red fluorescence. Prior to NPs stimulation, cells were washed with PBS twice and incubated with JC-1 staining reagent (1:1000) in medium at 37 °C for 30 min, followed by washing with PBS and cell treatment. The mitochondrial membrane potential was detected by fluorescence microscopy at time intervals of 10 min.

### Measurement of [Ca^2+^]_c_

All experiments were performed in dark conditions. The cells were loaded with a Rhod-2 AM dye (1 μM) (*K*_d_ = 570 nM, λ_Ex_ = 552 nm, and λ_Em_ = 581 nm) (Invitrogen, CA, USA) for 45 min. The cells were then washed with PBS twice before being incubated with Hanks buffer, and treated with the appropriate NPs concentration. All Ca^2+^ signaling experiments were carried out in a thermo-regulated state at 37 °C mounted on a Nikon microscope (Nikon Eclipse TE2000-U, Tokyo, Japan) [24, 25, 27] (Chen et al. 2011).

### Mucin Secretion and ELLA

The cells were seeded at 1 × 10^6^ cells per well in a 6-well plate and cultured for 24 h. Trachea primary cells were then rinsed with PBS and stimulated for 15 min with the corresponding LSM NP concentrations (500 μg/ml, 250 μg/ml, and 100 μg/ml) prepared in PBS. The supernatant containing secreted mucin was collected and briefly centrifuged at 8000 rpm to remove the residual NPs. The supernatant was then incubated in a 96-well (Nunc MaxiSorp, VWR, CA, USA) plate overnight at 4 °C. Afterward, the 96-well plate was washed with PBST (PBS + 0.05% Tween-20) and then blocked with 1% BSA. The 96-well plate was washed again with PBST and incubated with lectin (Wheat germ agglutinin, WGA) (Sigma-Aldrich, MO, USA), conjugated to horseradish peroxidase (HRP; 5 mg/ml) (Sigma-Aldrich, MO, USA), at 37 °C for 1 h. The substrate, 3,3′,5,5′-tetramethylbenzidine (TMB; Sigma-Aldrich, MO, USA), was added to each well at room temperature, followed by H_2_SO_4_ (Sigma-Aldrich, MO, USA) in order to terminate the reaction. The optical density was measured at 450 nm (Chen et al. 2011; Kemp et al. 2004).

### Immunosorbent Assay (ELISA) Preparation

The cells were seeded at a density of 1 × 10^6^ cell density in a 6-well plate and cultured for 24 h. Trachea cells were then rinsed with PBS. Cells were stimulated for 2 h with the appropriate LSM NPs concentrations (0–500 μg/ml) prepared in PBS. Cell lysing was prepared by the Peirce RIPA cell lysate reagent, and the lysate was collected and transferred to a microcentrifuge tube. The samples were centrifuged at ~ 14,000×*g* for 15 min to pellet the cell debris and NPs. The supernatant was then incubated in a 96-well plate overnight at 4 °C. Afterward, the 96-well plate was washed with PBST (PBS + 0.05% Tween-20) and then blocked with 1% BSA. The 96-well plate was washed again with PBST and incubated with rabbit anti-caspase 3, active form antibody (Millipore, polyclonal antibody) and mouse anti-cytochrome C (Invitrogen, monoclonal antibody) at room temperature for 2 h. Then use secondary antibody (anti-rabbit and anti-mouse conjugated horseradish peroxidase, HRP, Millipore) and followed by the same procedure as ELLA to measure the absorbance intensity [25].

### Image Analysis

Image analysis was performed with an inverted Nikon Eclipse TE2000-U fluorescent microscope. Each photo was taken at × 10 magnification and analyzed using Simple PCI (Compix Inc., Imaging Systems, Sewickle, PA, USA). The data shown for cytosolic calcium concentrations are represented by Rhod-2 fluorescence. Images were taken every 0.5 s and automatically converted to grayscale for analysis. Simple PCI provided the pixel intensities (mean gray value) of selected areas, each with a mean fluorescence per frame for 200 cells over 100 s (~ 200 frames) immediately following nanoparticles stimulation. The data shown for immunofluorescence staining is a representation of protein expression after 1–2 h treatments of graphene. All experiments were conducted and corroborated independently at least three times.

### Statistical Analysis

The data were presented as mean ± SD. Each experiment was performed independently at least three times. Statistical significance was determined using one-way ANOVA test analysis with *p* values < 0.05 (GraphPad Prism 4.0, GraphPad Software, Inc., San Diego, CA, USA).

## Abbreviations

BPE: Bovine pituitary extract
ELISA: Enzyme-linked immunosorbent assay
LSM: Lanthanum strontium manganite
ROS: Reactive oxygen species
SFM: Serum free medium
SOFCs: Solar oxidized fuel cells
